# An impedance threshold device did not improve carotid blood flow in a porcine model of prolonged cardiac arrest

**DOI:** 10.1186/s12967-020-02264-5

**Published:** 2020-02-14

**Authors:** Benedict Kjaergaard, Hans O. Holdgaard, Sigridur O. Magnusdottir, Søren Lundbye-Christensen, Erika F. Christensen

**Affiliations:** 1 Department of Cardiothoracic Surgery, Aalborg University Hospital, Hobrovej 18-22, 9100 Aalborg, Denmark; 2 Institute of Clinical Medicine, Aalborg University, Aalborg, Denmark; 3 Biomedical Research Laboratory, Aalborg University Hospital, Aalborg, Denmark; 4 Center for Prehospital and Emergency Research, Aalborg University and Aalborg University Hospital, Aalborg, Denmark; 5 Unit of Clinical Biostatistics, Aalborg University Hospital, Aalborg, Denmark; 6 Clinic of Medicine and Emergency Care, Aalborg University Hospital, Aalborg, Denmark

**Keywords:** Impedance threshold device, Cardiac arrest, Carotid blood flow

## Abstract

**Background:**

An impedance threshold device (ITD) was developed to increase venous return to the heart and therefore increase cardiac output and organ blood flow during cardiopulmonary rescue (CPR). Basic CPR aims to maintain coronary and cerebral blood flow at the minimum level necessary for survival. The present study compared the effects of an ITD on cerebral blood flow assessed as blood flow in both carotid arteries to the blood flow of a control group during prolonged CPR.

**Methods:**

Fourteen anaesthetized pigs were monitored during 60 min of CPR after induced ventricular fibrillation. The primary outcome was blood flow in both carotid arteries, and the secondary outcomes were blood pressure, acid–base parameters, plasma potassium, and plasma lactate. The pigs were randomized to mechanical compressions and ventilation with an ITD added to the ventilation or to a control group treated only with mechanical compressions and ventilation. The time course for the parameters was tested using analysis of variance.

**Results:**

The cumulative carotid blood flow in the ITD group decreased from 64 to 42 ml/min, and it decreased from 69 to 51 ml/min in the control group during 60 min of CPR. The difference was not significant. The secondary outcome measures were also not significantly different.

**Conclusions:**

This study did not show any beneficial effect of an ITD on carotid blood flow.

## Background

It is often necessary to perform prolonged cardiopulmonary resuscitation (CPR) on patients with cardiac arrest (CA) during transportation to a hospital. Under these conditions, it is helpful to use mechanical chest compression devices, such as LUCAS and Autopulse [1, 2]. Blood flow is limited even during the best-performed manual CPR, but these devices improve organ perfusion. Some studies have indicated that blood flow may be even better if a device such as the LUCAS is combined with an impedance threshold device (ITD), as described in porcine models [3–6]. The ITD is a valve connected to a cuffed tracheal tube. The device limits air entry into the lungs during chest recoil between chest compressions. The effect is a decrease in intrathoracic pressure and an increase in venous return to the heart. The manufacturer of an ITD, ResQPod (Advanced Circulatory Systems, Roseville, MN, USA), has a homepage where one of the references demonstrated that an ITD increased blood flow to the brain by 50% in porcine models [5]. However, other animal studies did not show an effect of an ITD on coronary perfusion pressure [7, 8]. Clinical studies in patients have shown varying results. In a blinded, randomized study with ITD or sham ITD in patients with out-of-hospital cardiac arrest, there was no difference in survival to hospital discharge [9]. Another study showed a benefit of an ITD but only in cases where the quality of CPR was good, and the opposite result was found if the patient did not receive an acceptable quality of CPR [10]. According to the 2015 European Resuscitation Council guidelines [11] and the 2015 American Heart Association guidelines [12], an ITD is not recommended for routine use in CPR. However, it is of clinical interest to know if an ITD improves brain circulation during prolonged CPR when DC conversions and medications do not result in return of spontaneous circulation (ROSC). Under these conditions, another method with extracorporeal life support may be a bridge to diagnosis, treatment and survival provided that the circulation to the brain was sufficient until ECC was established [13, 14].

The present porcine study examined the effects of an ITD on the cumulated blood flow in the internal carotid arteries during CPR. Other studies used only one carotid artery with a similar purpose [4, 15].

## Methods

The Danish Animal Experiments Inspectorate approved the experiment (no. 2016-15-0201-00866), which was performed in accordance with the Utstein-style guidelines for reporting laboratory cardiopulmonary resuscitation [16].

Study design: Randomized controlled experimental animal study. The statistician performing the analysis was blinded to the randomization.

### Animal care

Fourteen female Danish Landrace pigs were used for this study. The animals were allowed 1 week of acclimation after arrival to the research facility. They were housed in pairs, with free access to water and feed.

The pigs were pre-anaesthetised with an intramuscular injection of a Zoletil (Virbac, Kolding, Denmark) mixture containing two dissociative medications (ketamine 8.3 mg/ml and tiletamine 8.3 mg/ml), a benzodiazepine (zolazepam 8.3 mg/ml), an opioid (butorphanol 1.7 mg/ml), and xylazine (8.3 mg/ml). Anaesthesia was maintained with a continuous infusion of propofol and fentanyl until ventricular fibrillation (VF) was induced, when all medications were withdrawn. A single dose of 2.500 IU heparin was given to inhibit blood clots in the vascular catheters. No vasopressors or other medications were used during the 1-h study period.

The animals were paralyzed with 50 mg of rocuronium prior to VF induction to inhibit a confounding effect of gasping [17].

The trachea was intubated with a 6.5-mm endotracheal tube, and the lungs were mechanically ventilated with a ventilator (Dameca Dream, Roedovre, Denmark). The tidal volume (TV) was 10 ml/kg, and the positive end-expiratory pressure (PEEP) was 5 cm H_2_O. The fraction of inspired oxygen (FiO_2_) was 0.6 during the surgical procedures. The TV was higher than recommended in guidelines for humans, but it is the normal TV range for pigs [18, 19]. The respiratory rate (RR) was adjusted to keep the partial pressure of carbon dioxide in arterial blood (PaCO_2_) at 4.5–5.5 kPa with an unaltered TV because a change in TV could influence the effect of an ITD.

A central 7 Fr venous catheter was inserted via the right jugular vein to the level of the right atrium for drug and fluid administration and VF induction. A 6 Fr femoral catheter was inserted into the right femoral artery for continuous blood pressure monitoring and blood tests.

Electrocardiography (ECG), bladder temperature, end tidal CO_2_ (ETCO_2_), and blood pressure (BP) were monitored continuously using a Propac MD monitor (Zoll, Medidyne, Copenhagen, Denmark).

One litre of saline was infused during instrumentation to rehydrate the animals after fasting prior to the experiments.

A bladder catheter with a temperature gauge (Degania Silicone Ltd. Degania Bet 15130, Israel) was inserted for urine drainage and temperature monitoring.

The animal’s temperature was kept at a level of normothermia using a warming blanket, if needed.

Both internal carotid arteries were exposed, and a perivascular ultrasound transit time flow probe of 4 mm (Medi-Stim ASA, Fernanda Nissensgate 3, Oslo, Norway) was fitted on the arteries to measure blood flow. The combined flow in the two arteries was considered a reflection of the blood flow to the brain.

The animals were euthanized with an overdose of pentobarbital at the end of the study period.

### Experimental procedures

Before initiating VF, FiO_2_ was reduced to 0.23, which is the lowest possible set point for the ventilator, and induction of VF was not performed if PaO_2_ was above 15 kPa.

**Fig. 1:**
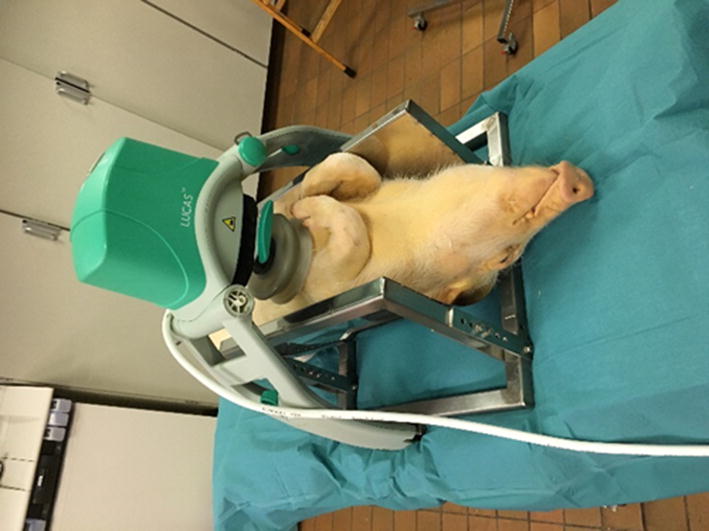
Photo of the homemade pig holder showing the animal slightly turned to the right side to avoid injuries from the Lucas1 Cardiac Compression Device

VF was induced with a pacemaker wire inserted via the central venous catheter into the right ventricle using a 9-V DC shock. CA was defined as VF and systolic BP below 25 mmHg according to the Utstein guidelines [16]. Just after induction of VF, the ventilator was adjusted to a FiO2 of 1.0, and RR was set to 10/min with unaltered tidal volume but with no PEEP. Mechanical cardiac compressions were started immediately using LUCAS1™ at 100 compressions/min, a compression:decompression relationship of 1:1 and a compression depth of 5 cm [1]. The animals were placed in a homemade pig holder for the LUCAS device (LUCAS 1, Jolife AB, Ideon Science Park, SE-223 70 Lund, Sweden) to keep the pigs securely positioned and slightly turned to the right side, which in our experience results in fewer injuries to the pig during cardiac compressions (Fig. 1).

On each of the seven study days, 2 pigs were randomized to one of the 2 groups immediately after VF. Thus, seven pigs were randomized to each group.

#### ITD group

Ventilation was continued using the ventilator with the same settings as described for the control group, but an ITD (ResQPod, Advanced Circulatory Systems, Roseville, MN, USA) was connected to the tube as an interface between the animal and the ventilator to inhibit air flow into the trachea during the decompression phase.

#### Control group

Ventilation was continued using the ventilator with an unchanged TV of 10 ml/kg and an RR of 10/min; PEEP was reduced to zero, and FiO_2_ was increased to 1.0.

Cardiac compressions for both groups were started immediately following VF induction using the LUCAS1 device at a frequency of 100/min and were continued for 60 min.

### Exclusion criteria

The Utstein recommendations for animal experiments of resuscitation define circulatory arrest as a systolic BP below 25 mmHg [16]. However, since our primary outcome was carotid blood flow during 60 min of mechanical CPR, data were included even if BP decreased below 25 mmHg. The experiment was terminated if an animal had ROSC.

### Experimental outcomes

The primary outcome measure was the change in blood flow in both carotid arteries after 60 min compared to the flow 5 min after the induction of cardiac arrest. The secondary outcome measures were systolic BP, pH, PaO_2_, ETCO_2_, plasma potassium and plasma lactate.

### Measurements

The following values were measured just prior to VF induction (i.e., precardiac arrest): total blood flow in both carotid arteries (carotid BF), systolic femoral blood pressure (BP), and ETCO_2_. Blood gas analysis was performed using an ABL 800 Radiometer (Copenhagen, Denmark). pH, PaO_2_, potassium and lactate concentration values are presented in the results.

Bladder temperature was continuously measured, and the temperature was kept in the normal range using warming blankets.

All measurements were performed every 5 min after VF induction during the period of investigation up to 1 h. Cardiac compressions were paused for 5 s during each measurement to evaluate ROSC.

### Statistics

Distributions are described as the means and standard deviations (SDs). Comparisons of baseline distributions between treatment groups were performed using an unpaired t-test.

To describe the time course in the two groups, spline curves were fitted to the time series data of outcome variables by means of repeated measures one-way analysis of variance (ANOVA). The levels of outcome variables at time t = 5 and t = 60 were estimated from these splines. In all analyses, bootstrapping with 10.000 replications was used for calculations of standard errors, and p-values were used to account for potential violations of assumed normality and correlation structure in the data.

A value of p < 0.05 was regarded as statistically significant.

Stata Version 15 (Stata Corporation, College Station, TX, USA) was used for all calculations and graphs.

## Results

**Fig. 2:**
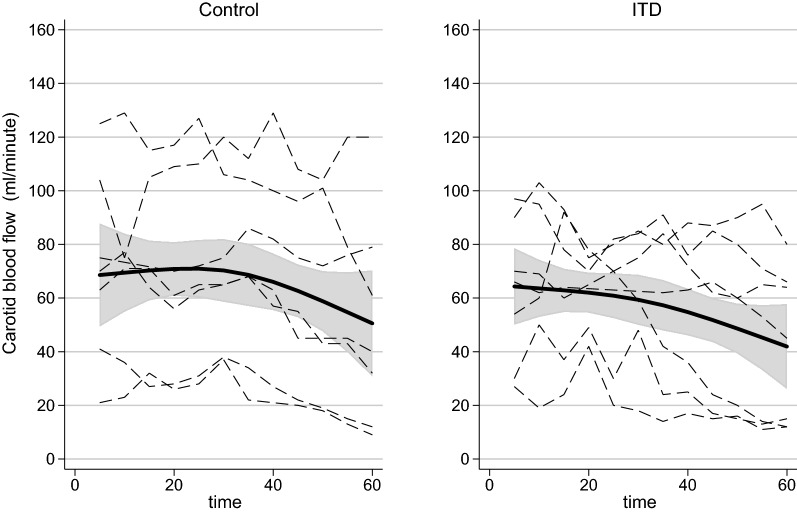
Graph showing smooth ANOVA spline curves and 95% Bayesian confidence lines for carotid blood flow for animals in the control group and in the ITD from time + 5 min to 60 min following cardiac arrest

**Fig. 3:**
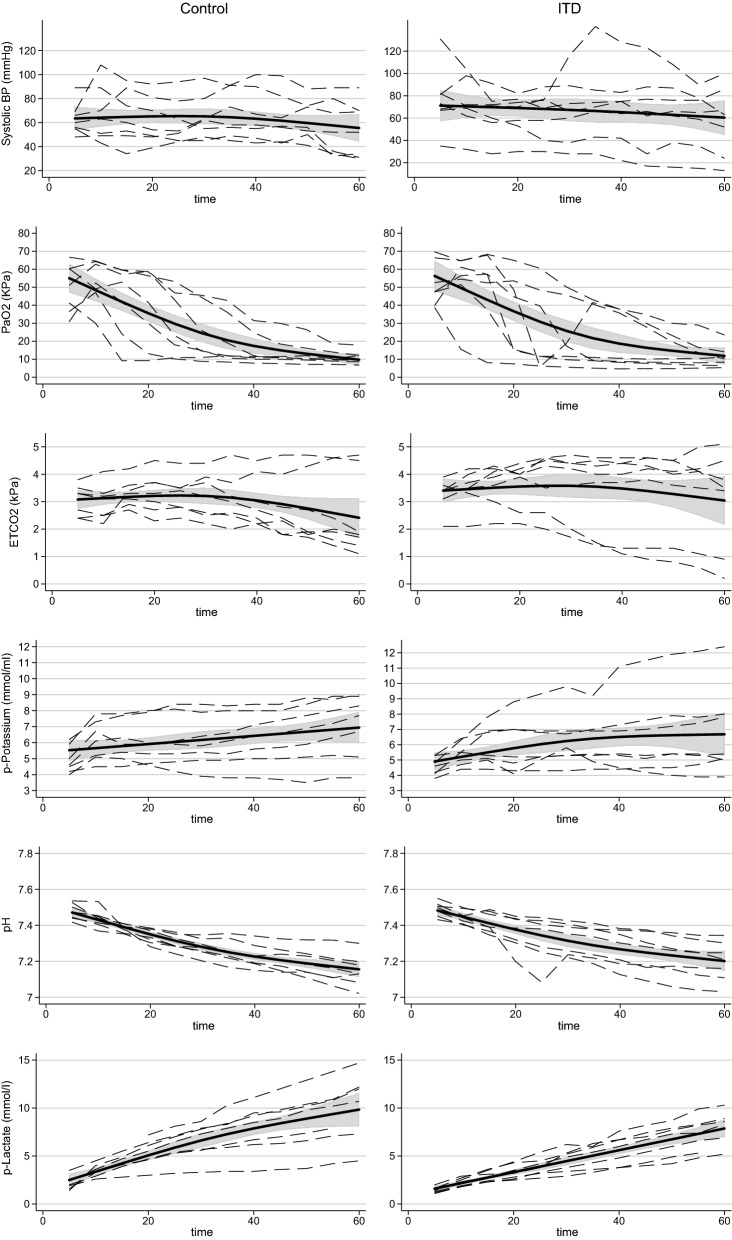
Graphs showing smooth ANOVA spline curves and 95% Bayesian confidence lines for blood pressure, pH, PaO_2_, ETCO_2_, and P-potassium for animals in the control group and in the ITD group from time + 5 min to 60 min following cardiac arrest

Pre-CA values are indicated in Table 1. The ITD group had a significantly higher flow in the carotid arteries (446 ml/min) vs. the control group (280 ml/min). There was also a very small but significant difference in pH, which was 7.47 in the ITD group and 7.43 in the control group. Both baseline pH values were in the normal range for pigs. There was no flow or pH difference at the first measurements at CA + 5 min, as indicated in Table 2, but there was a significantly higher p-lactate in the control group after 5 min of CA. This difference disappeared after 60 min of CA. Table 3 shows the measured values at the end of the study after 60 min of CA. These exact values at the end of the study are of interest, but the smooth ANOVA spline curves over time are even better to show the time course, as shown in Figs. 2 and 3.

**Table 1:** Pre-cardiac arrest values for carotid blood flow, systolic blood pressure, pH, PaO_2_, ETCO_2_, plasma potassium, and plasma lactate

Group	ITD group, N = 7	Control group, N = 7	p values
Carotid blood flow (ml/min)	446 (106)	280 (106)	0.003
Blood pressure (mmHg)	131 (24)	133 (24)	0.833
PaO_2_ (kPa)	13.4 (1.9)	12.4 (1.3)	0.228
ETCO_2_ (kPa)	4.9 (0.7)	5.0 (0.4)	0.615
pH	7.47 (0.03)	7.43 (0.04)	0.015
p-Potassium (mmol/l)	3.76 (0.29)	4.04 (0.68)	0.286
p-Lactate (mmol/l)	1.11 (0.31)	1.37 (0.66)	0.329

The values are the means with standard deviation

**Table 2:** First measured values after 5 min of cardiac arrest for carotid blood flow, systolic blood pressure, pH, PaO_2_, ETCO_2_, plasma potassium, and plasma lactate

Group	ITD group, N = 7	Control group, N = 7	p values
Carotid blood flow (ml/min)	62.0 (27.1)	71.3 (35.3)	0.566
Blood pressure (mmHg)	76.7 (28.6)	63.3 (13.3)	0.245
PaO_2_ (kPa)	51.7 (12.1)	49.6 (13.7)	0.748
ETCO_2_ (kPa)	3.3 (0.6)	3.1 (0.6)	0.545
pH	7.49 (0.04)	7.48 (0.05)	0.549
p-Potassium (mmol/l)	4.59 (0.59)	4.91 (0.84)	0.379
p-Lactate (mmol/l)	1.44 (0.30)	2.13 (0.75)	0.026

The values are the means with standard deviation

**Table 3:** Last measured values after 60 min of cardiac arrest for carotid blood flow, systolic blood pressure, pH, PaO_2_, ETCO_2_, plasma potassium, and plasma lactate

Group	ITD group, N = 7	Control group, N = 7	p values
Carotid blood flow (ml/min)	42 (29)	50 (40)	0.638
Blood pressure (mmHg)	58 (31)	54 (23)	0.763
PaO_2_ (kPa)	11.3 (6.1)	10.9 (3.6)	0.873
ETCO_2_ (kPa)	3.1 (1.8)	2.4 (1.5)	0.477
pH	7.20 (0.11)	7.15 (0.09)	0.318
p-Potassium (mmol/l)	6.80 (2.90)	7.07 (1.99)	0.833
p-Lactate (mmol/l)	8.03 (1.77)	10.23 (3.70)	0.177

The values are the means with standard deviation

All seven animals in the ITD group and all seven animals in the control group were included in all 60 min of CA.

Two animals in the ITD group experienced a drop in BP below 25 mmHg, one animal after 35 min and the other animal after 55 min, but as mentioned, the measurements continued for all seven animals for the 60 min of CA.

### Cumulative flow in the internal carotid arteries

The measured mean values between the induction of CA + 5 min and at the end after 60 min are shown in Tables 2 and 3. The cumulative blood flow in the carotid arteries decreased during the study period from 64.3 to 41.9 ml/min in the ITD group, as estimated from the spline curves (Fig. 2), compared to 68.6 to 50.6 ml/min in the control group. There was no significant difference between the groups after 5 min of CA or after 60 min of CA (Tables 2 and 3). The shape of the time course during the study period did not differ between the treatment groups (p = 0.99).

### Blood pressure

Systolic BP in the group treated with ITD decreased from 71.2 to 60.4 mmHg. Systolic BP in the control group decreased from 63.5 to 55.5 mmHg. There was no significant difference between the groups after 5 min of CA or after 60 min of CA (Tables 2 and 3). The shape of the time course during the study period did not differ between the treatment groups (p = 0.98).

### Partial pressure of oxygen

The decrease in PaO_2_ in the ITD group was from 56.3 to 11.9 kPa, and in the control group, the decrease was from 55.1 to 9.7 kPa. There was no significant difference between the groups after 5 min of CA or after 60 min of CA (Tables 2 and 3). The shape of the time course during the study period did not differ between the treatment groups (p = 0.99).

### ETCO_2_

ETCO_2_ in the ITD group decreased from 3.4 to 3.0 kPa. ETCO_2_ in the ITD group decreased from 3.1 to 2.4 kPa. There was no significant difference between the groups after 5 min of CA or after 60 min of CA (Tables 2 and 3). The shape of the time course during the study period did not differ between the treatment groups (p = 0.98).

### P-potassium

P-potassium in the ITD group increased from 4.9 to 6.7 mmol/l, and the increase in the control group was from 5.3 to 6.9 mmol/l. There was no significant difference between the groups after 5 min of CA or after 60 min of CA (Tables 2 and 3). The shape of the time course during the study period did not differ between the treatment groups (p = 0.21).

### pH

The pH decreased from 7.48 to 7.20 in the ITD group, and in the control group, the pH decreased from 7.47 to 7.16. The minimal difference in baseline pH disappeared, and there was no significant difference between the groups after 5 min of CA or after 60 min of CA (Tables 2 and 3). The shape of the time course during the study period did not differ between the treatment groups (p = 0.58).

### P-lactate

P-lactate increased from 1.60 to 7.87 in the ITD group, and in the control group, p-lactate increased from 2.50 to 9.84. There was a significantly lower p-lactate level in the ITD group after 5 min of CA, but the difference was not significant after 60 min of CA (Tables 2 and 3). The shape of the time course during the study period did not differ between the treatment groups (p = 0.17).

## Discussion

The present study did not show any superior effects on cerebral blood flow or acid–base parameters in a group of pigs with CA treated with an ITD compared to a control group during 1 h of CPR. The differences in the baseline measurements of cerebral blood flow could not be explained by anything other than coincidence, and even though the differences appeared to be in favour of the ITD group, there was no difference during the whole period of CA.

Reports on the use of an impedance threshold device are conflicting. More animal studies have indicated beneficial effects on haemodynamic parameters, such as increased coronary perfusion pressure and cerebral perfusion pressure [3–6], and one of these showed improved 24- and 48-h survival and neurological outcomes [6]. Other studies did not indicate any positive effects on haemodynamic parameters [7, 8]. The present study could not evaluate any possible beneficial effect on the haemodynamics during a shorter duration of CPR, which could influence the possibility of ROSC after DC conversion. In clinical practice, DC is often tried as early as possible. The relatively stable EtCO_2_ in both groups during 60 min of CPR indicates that the quality of CPR was good.

It was not possible to measure blood flow in the vertebral arteries, which also supply the cerebrum with blood, but we assumed that the blood flow in the carotid arteries reflected the total cerebral blood flow. This assumption is consistent with a previous study that measured blood flow to the brain by measuring cerebral blood flow using an injection of radioactively labelled microspheres and a peripheral flow probe placed on the right common carotid artery [4]. The researchers demonstrated an equivalent decrease in cerebral blood flow using both methods. Debaty et al. [3] demonstrated no increase in cerebral blood flow using a flow probe on the left carotid artery. In contrast, Lurie et al. [5] demonstrated a 20% increase in cerebral blood flow (from 0.19 to 0.23 ml/g/min) during CPR combined with ITD, also using a technique involving the injection of radiolabelled microspheres.

The conflicting results support the need for more research, especially because medical equipment is not subjected to the same regulations as new medical drugs, which need approval by government authorities, such as the US Food and Drug Administration, European Commission or other national authorities.

### Strengths

The design of the present study allowed for the creation of a prolonged and controlled cardiac arrest period to test our hypothesis that an ITD would increase blood flow to the brain during the study period of 1 h. It was possible to measure blood flow continuously in both carotid arteries. The statistician was blinded to the study results.

### Limitations

The major limitation is the transition from animal to human studies due to the species differences between humans and pigs. The LUCAS device is for human use, but it was originally designed for studies in pigs. The present study did not evaluate the effect of ITD on haemodynamics, such as central venous return or coronary perfusion pressure.

The entire study group was not blinded.

## Conclusion

In conclusion, the present study did not show any beneficial effects of an ITD on the cumulative carotid blood flow or acid–base parameters in pigs with VF treated with CPR for 1 h.

## Data Availability

The data are available from the corresponding author on reasonable request.

## Abbreviations

ANOVA: Analysis of variance
BP: Blood pressure
CA: Cardiac arrest
CPR: Cardiopulmonary resuscitation
ECG: Electrography
EtCO_2_: End tidal carbon dioxide
FiO_2_: Inspired fraction of oxygen
ITD: Impedance threshold device
TV: Tidal volume
VF: Ventricular fibrillation
